# Raspberry Pi nest cameras: An affordable tool for remote behavioral and conservation monitoring of bird nests

**DOI:** 10.1002/ece3.8127

**Published:** 2021-10-11

**Authors:** Hannah F. R. Hereward, Richard J. Facey, Alyssa J. Sargent, Sara Roda, Matthew L. Couldwell, Emma L. Renshaw, Katie H. Shaw, Jack J. Devlin, Sarah E. Long, Ben J. Porter, Jodie M. Henderson, Christa L. Emmett, Laura Astbury, Luke Maggs, Sean A. Rands, Robert J. Thomas

**Affiliations:** ^1^ Cardiff School of Biosciences Cardiff University Cardiff UK; ^2^ Department of Biology University of Washington Seattle Washington USA; ^3^ A Rocha, Cruzhina Alvor Portugal; ^4^ Gypseywood Cottage York UK; ^5^ University of Cambridge Cambridge UK; ^6^ University of Kentucky Lexington Kentucky USA; ^7^ Tan y Garn Rhiw UK; ^8^ Department of Applied Sciences University of the West of England Bristol UK; ^9^ D3 Data Driven Decisions Cardiff UK; ^10^ School of Biological Sciences University of Bristol Bristol UK

**Keywords:** Animal behavior, bespoke camera, burrow‐nesting, interspecific interactions, nest box, Raspberry Pi, seabirds, storm‐petrel

## Abstract

1. Bespoke (custom‐built) Raspberry Pi cameras are increasingly popular research tools in the fields of behavioral ecology and conservation, because of their comparative flexibility in programmable settings, ability to be paired with other sensors, and because they are typically cheaper than commercially built models.
2. Here, we describe a novel, Raspberry Pi‐based camera system that is fully portable and yet weatherproof—especially to humidity and salt spray. The camera was paired with a passive infrared sensor, to create a movement‐triggered camera capable of recording videos over a 24‐hr period. We describe an example deployment involving “retro‐fitting” these cameras into artificial nest boxes on Praia Islet, Azores archipelago, Portugal, to monitor the behaviors and interspecific interactions of two sympatric species of storm‐petrel (Monteiro's storm‐petrel *Hydrobates monteiroi* and Madeiran storm‐petrel *Hydrobates castro*) during their respective breeding seasons.
3. Of the 138 deployments, 70% of all deployments were deemed to be “Successful” (Successful was defined as continuous footage being recorded for more than one hour without an interruption), which equated to 87% of the individual 30‐s videos. The bespoke cameras proved to be easily portable between 54 different nests and reasonably weatherproof (~14% of deployments classed as “Partial” or “Failure” deployments were specifically due to the weather/humidity), and we make further trouble‐shooting suggestions to mitigate additional weather‐related failures.
4. Here, we have shown that this system is fully portable and capable of coping with salt spray and humidity, and consequently, the camera‐build methods and scripts could be applied easily to many different species that also utilize cavities, burrows, and artificial nests, and can potentially be adapted for other wildlife monitoring situations to provide novel insights into species‐specific daily cycles of behaviors and interspecies interactions.

## Graphical abstract

We present a methodological paper focusing on a practical issue, describing a novel and easily constructed field camera system that has been designed (and rigorously tested) for deployment in remote locations with harsh conditions. We describe how the system can be easily adapted for diverse conservation/management and field ecology projects.

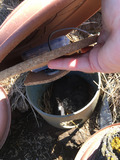

## 1 INTRODUCTION

The use of photography and video systems to remotely monitor wildlife has become increasingly popular (see reviews: Cutler & Swann, 1999; Edney & Wood, 2020; Hereward et al., Under review; Swann et al., 2004; Trolliet et al., 2014). This is because remote‐monitoring cameras can greatly reduce the time and effort required to collect observational field data and are typically less invasive than direct observation by researchers in the field (Cutler & Swann, 1999; Trolliet et al., 2014). However, designing, implementing, and maintaining camera systems can require technical expertise; the presence of the camera can potentially affect an animal's behavior; and the type of data collected can be limited (Caravaggi et al., 2020; Cutler & Swann, 1999; Reif & Tornberg, 2006; Trolliet et al., 2014). Nevertheless, infrared‐sensitive, movement‐triggered video cameras now enable greater flexibility than earlier designs in remote surveillance of wildlife (Scheibe et al., 2008), and videomonitoring has increasingly been used to aid population monitoring and to examine behavioral and ecological interactions (Meek et al., 2014; Trolliet et al., 2014).

There are a wide range of camera systems available (see reviews: Cutler & Swann, 1999; Edney & Wood, 2020; Hereward et al., Under review; Swann et al., 2004; Trolliet et al., 2014), but these can be split broadly into (a) commercially (vendor) built systems (e.g., Meek & Pittet, 2012; Trolliet et al., 2014) or (b) bespoke (user‐built) microcomputer systems (Allan et al., 2018; Greenville & Emery, 2016; Johnston & Cox, 2017).

Commercially built systems are typically easier to use, with little setup time or knowledge of the system required (Cox et al., 2012; Hereward et al., Under review; Meek & Pittet, 2012). However, their deployment settings are typically less flexible, specifically in the length of time cameras can be left during deployments due to limited battery life and image/footage storage capabilities, and due to the limited programmable settings available (Cox et al., 2012; Prinz et al., 2016; Reif & Tornberg, 2006). By contrast, simple programmable computers, or circuit boards, such as Raspberry Pi (www.raspberrypi.org) or Arduino (www.arduino.cc), have been increasingly used by researchers (Hereward et al., Under review). These technologies have allowed greater scope for the development of purpose‐built cameras and for addressing specific research questions (Allan et al., 2018; Greenville & Emery, 2016; Johnston & Cox, 2017; Jolles, 2021). The increasing popularity of these bespoke units is not only driven by their comparative flexibility in programmable settings, but also by the reduced costs and by the cameras being combined with other sensors, for example, temperature loggers (McBride & Courter, 2019). Do‐it‐yourself, self‐assembly cameras can be produced more cheaply than commercially available models; for example, Cox et al. (2012) calculated that their bespoke system (“System One”) costs ~33% less than a comparable prebuilt unit. However, it is important to note that these bespoke cameras require additional expertise and time to design, setup, and troubleshoot (Cox et al., 2012; Hereward et al., Under review).

Raspberry Pi has been used as the foundation to develop bespoke units to study a variety of taxa (see recent reviews: Hereward et al., Under review; Jolles, 2021), including video monitoring of free living fish (Mouy et al., 2020); laboratory studies of fish behaviors (Jolles et al., 2018); in situ Lemming (*Lemmus* spp. and *Dicrostonyx* spp.) subnival behaviors (Kalhor et al., 2019); behavior, surface body temperature, and respiration rate of hibernating meadow jumping mice (*Zapus hudsonius*) (Kallmyer et al., 2019); behaviors of captive song birds (Alarcón‐Nieto et al., 2018); behaviors of birds at baited traps (Nazir, Newey, et al., 2017); behavioral dynamics and interindividual/interspecific interactions at bird feeders (McBride & Courter, 2019; Youngblood, 2020); and breeding behaviors of cavity‐nesting birds (Prinz et al., 2016).

Some of these papers specifically describe the building methods of the camera setup, where the costs ranged from ~$85 USD (Youngblood, 2020) to ~1,000€ (Zárybnická et al., 2016). A range of different power sources were used: (a) mains power or large batteries (60 Ah 12 V battery), occasionally attached to solar panels, providing power lasting 6.5–7 days (Nazir, Newey, et al., 2017; Prinz et al., 2016; Zárybnická et al., 2016); (b) smaller powerpacks of 10,000–20,000 mAh often attached to solar panels lasting 4–7 days (McBride & Courter, 2019; Youngblood, 2020); and (c) D‐cell batteries in series, creating 70,000 mAh, which lasted at least 14 days (Mouy et al., 2020). For storing the recorded image/video files, various designs coded the Raspberry Pi to upload the files from the SD card to “the cloud”, thus avoiding the need to remove the SD card periodically and reducing the likelihood of the SD card becoming full (Alarcón‐Nieto et al., 2018; McBride & Courter, 2019; Prinz et al., 2016; Youngblood, 2020; Zárybnická et al., 2016). However, Mouy et al. (2020) were not able to connect their system to a network during deployment and so found that their SD card capacity (200 GB) became the limiting factor for storage over the 8–14 days that their devices were deployed, recording a maximum of 212 hr. However, Mouy et al. (2020) found that during trials, using USB storage rather than SD storage used more energy, therefore reducing battery life. USB storage was also less reliable, due to having a more fragile connection, for example, vibrations from the boat disrupting the connection prior to deployment (Mouy et al., 2020). In comparison, Kallmyer et al. (2019) successfully used a 32 GB USB for data storage. Regarding cameras, only Youngblood (2020) did not use a camera, but instead paired passive integrated transponders on the birds, with a radiofrequency identification reader at the feeders. The rest of these studies used a variety of different camera types including Pi NoIR (Kallmyer et al., 2019; Nazir, Newey, et al., 2017; Prinz et al., 2016) or Raspberry Pi camera module v2 (Alarcón‐Nieto et al., 2018; Mouy et al., 2020), often combined with some form of passive infrared (PIR) detection system (Nazir, Newey, et al., 2017; Prinz et al., 2016; Zárybnická et al., 2016), or using changes in pixel intensity to indicate movement (Prinz et al., 2016).

There are a few published papers that detail the build of cameras to monitor cavity‐nesting species, using Raspberry Pi (Kalhor et al., 2019; Kallmyer et al., 2019; Prinz et al., 2016) or using a Linux FTP server control board (Zárybnická et al., 2016), including specifically for birds (Prinz et al., 2016; Zárybnická et al., 2016). All of these are designed so that the camera(s) (and additional modules) are embedded within—and become a part of—the nest box design. This is useful because the same nest box can be monitored over a long period. However, this is also restrictive in cases where the focal animals do not end up using the specific nest box, as happened for Prinz et al. (2016) due to changes in group composition. It also reduces the number of different nests monitored, compared to having the possibility of moving a camera system between nest boxes, which would allow greater insight into a wider number of nests/individuals across each breeding season.

Deploying cameras in extreme environments is technologically challenging due to the impact these conditions have on the performance and degradation of the equipment being used (O'Connell et al., 2011). However, several of the published camera systems have implemented waterproofing of the equipment. These deployments have included cold locations and therefore frosty conditions (Kalhor et al., 2019), as well as underwater (including marine) locations where not only does the case need to be watertight but also needs to cope with salt water and high water pressure (Greene et al., 2020; Mouy et al., 2020; Phillips et al., 2019).

For terrestrial systems, some camera systems would be completely exposed to rain, humidity and salt spray (if near the coast), and so mitigation has typically taken the form of water‐resistant/waterproof casings, for example, using a Peli case (peliproducts.co.uk) (Youngblood, 2020) or similar casing (e.g., Camacho et al., 2017; McBride & Courter, 2019), or a double box with drainage holes in the outer box (Nazir et al., 2017). However, other systems have been partially enclosed (e.g., a waterproof junction box; Prinz et al., 2016) due to being within a cavity/box and so less mitigation was deemed necessary, or not encased due to being fully enclosed within the nest box (e.g., Kalhor et al., 2019; Zárybnická et al., 2016). Nevertheless, despite the weather proofing of these terrestrial systems, humidity leading to condensation or frost on the camera lens still occurred with little additional mitigation suggested, other than removing or replacing the equipment (Camacho et al., 2017; Kalhor et al., 2019; Kallmyer et al., 2019), and including silica gel packets within the weatherproof casing during deployment (Youngblood, 2020).

Here, we describe a novel camera system that is fully portable and yet weatherproof, which was developed to study the behavior of two sibling species of sympatric, nocturnal, cavity‐nesting storm‐petrels (Hydrobatidae) that breed on Praia Islet, an isolated, uninhabited, volcanic islet (~12 ha) in the Azores archipelago, Portugal (Bolton et al., 2004; Long et al., in press). While there are now various bespoke camera models described in the scientific literature, few combine mitigation strategies for both salt spray and humidity alongside the need for easy access and full portability between nests throughout a single breeding season. Consequently, these unique circumstances presented by our study system required the development of a novel method of deployment. This included a bespoke camera and housing design to be fully portable between the 150 previously deployed artificial nest boxes on Praia Islet. These nest boxes were initially deployed in 2000, to provide additional breeding sites for two storm‐petrel species: the Monteiro's storm‐petrel *Hydrobates monteiroi* breeding in the “hot” season (April–September), and the Madeiran storm‐petrel *Hydrobates castro* breeding in the “cool” season (September–March) (Bolton et al., 2004, 2008; Bried et al., 2009). The camera system was required to record behaviors and interspecific interactions in these artificial nests over successive 24‐hr periods, on an isolated islet with no mains power supply, where it is difficult to bring in bulky equipment, and where the equipment would frequently be exposed to conditions of salt‐laden spray and high humidity. Here, we detail how this system can be deployed effectively in these circumstances (see appendices materials for full build details).

## 2 MATERIALS AND METHODS

**FIGURE 1:**
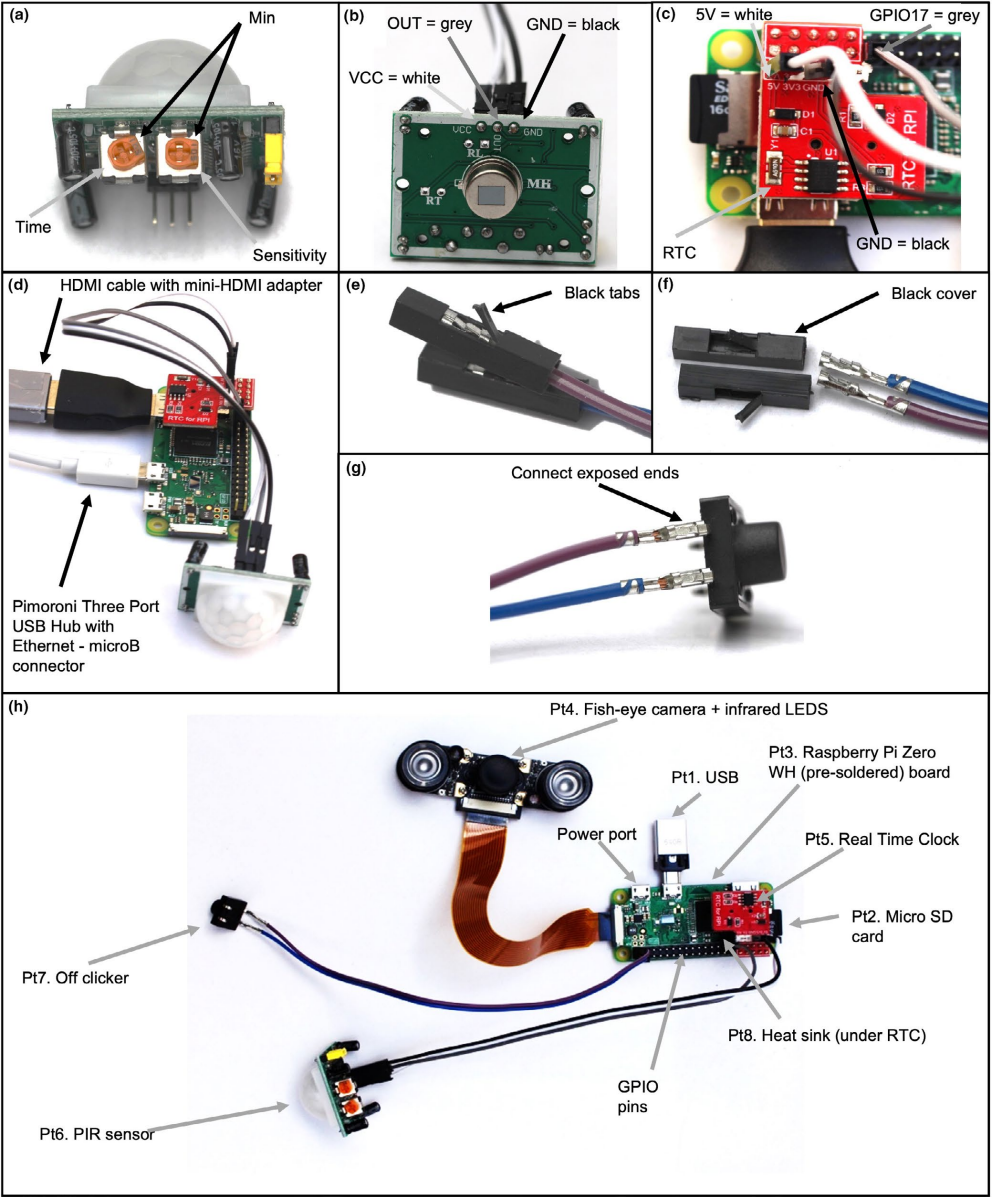
Pictures illustrating the building of the Raspberry Pi camera described in this study. (a) Passive infrared (PIR) sensor, showing the suggested positions of the sensor settings (sensors labeled with gray arrows, minimum (“min”) labeled with black arrows), the left setting =time (set at “min”) and the right setting = sensitivity (set at 90° to min); (b) PIR sensor without the sensor cover, showing the pin connections: white cable = VCC, gray = OUT, black = GND (labeled with respective arrows); (c) Real Time Clock (RTC) (red board, labeled with gray arrow) already connected to the Raspberry Pi board (GPIO pins 1–10), PIR sensor cables connecting onto the Real Time Clock 5V = white cable and GND = black and on the Raspberry Pi zero board, GPIO17 (pin 11) = gray (labeled with respective arrows); (d) completely connected Real Time Clock and PIR sensor, labeling the HDMI and USB connector ports; (e–g) to connect the switch to the Raspberry Pi board using two female–female cables, first remove the black covers on the switch end of the female–female cables by lifting the black tabs (e), then remove the black covers (f), finally attach to the switch by connecting the exposed ends of the female–female cable to two of the switch ends (g); and h) final built camera ready to be deployed labeled with each part

**FIGURE 2:**
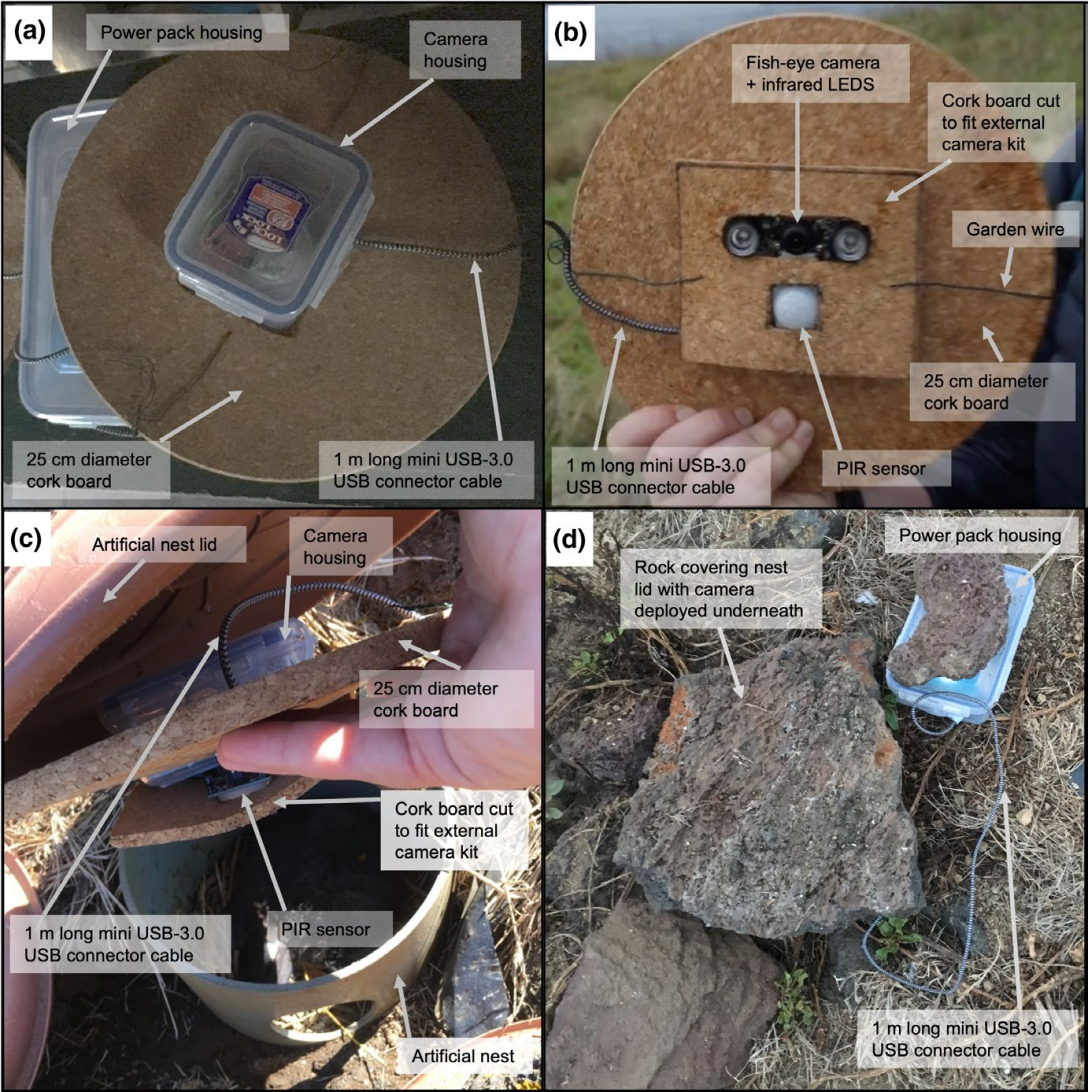
Photographs of the camera in various stages of deployment labeled with the different parts visible. (a) The top of the housing showing the camera housing, main cork board that sits on top of the nest box rim, power pack housing and the USB cord; (b) the underside of the housing with the main cork board again, this time showing the camera and PIR sensor which are held with the additional square of cork; (c) showing where the camera sits—on top of the nest box rim, below the nest box lid—and showing the different parts of the camera and (d) the camera deployed and hidden underneath a rock covering the artificial nest box, with powerpack + housing to the side with a rock on top to weigh the housing down. Deployed on Praia islet, Graciosa, Azores

We used a Raspberry Pi Zero circuit board, programmed using Python 3.5.3, paired with a fisheye camera with infrared LED attachments to create a bespoke camera small enough to fit on top of a storm‐petrel artificial nest box (Bolton et al., 2004; Figure 1, Figure 2) and programmed to record when triggered by a change in infrared levels (detected using a passive infrared [PIR] sensor). After triggering, recording lasted for 30 s with a 10‐s break between each recording. The resulting video files were stored on a USB flash dive (cf. McBride & Courter, 2019; Mouy et al., 2020). The camera housing was designed to be weather resistant through the use of plastic Tupperware containers, and silicon sealant was used around holes drilled for the wiring (Figure 2). Each camera cost a total of ~£86 GBP (~$115 USD) to build, with additional costs of ~£23 GBP (~$31 USD) per camera housing and ~£100 GBP (~$133 USD) needed for equipment to allow the construction of multiple cameras prior to deployment (Figures 1 and 2; see full part details and build methods in the Appendix A, with costs detailed in Table A1; deployment data and Python scripts archived with Dryad; Hereward et al., 2022).

### 2.1 Field deployment example

Fieldwork took place across the breeding seasons of both storm‐petrel species breeding on Praia Islet, Azores: Monteiro's storm‐petrel *H. monteiroi* (May–September 2019) and Madeiran storm‐petrel *H. castro* (early breeding season: September–early‐December 2019 and late breeding season: mid‐January–March 2020) (Praia Islet accessed under licence from Direção Regional do Ambiente, Região Autónoma dos Açores: SAI‐DRA/2019/1821 Proc. 116.14.03/43). Of the 150 artificial nest boxes available, nests were selected for videomonitoring based upon (a) whether the nest box was occupied, (b) accessibility of the nest box, and (c) whether the lid of the box was at an appropriate height above the nest (so that the footage captured would be in focus at a vertical distance of ≥15 cm). One camera per nest was deployed opportunistically across the subset of appropriate nests (*n* = 54) for 24 hr at a time, across the successive breeding seasons. During each 24‐hr deployment, at least two cameras were deployed in different nests. Each camera was removed after the 24‐hr period, the footage downloaded, and then, each camera was opportunistically re‐deployed at another nest of suitable breeding stage. The frequency of redeployments was dependent on the available (solar) power to charge the powerpacks.

In this paper, we present the technical outcomes, using a table of definitions, to define whether each of the deployments was a Failure, Partial failure (nonusable), Partial failure (usable), or a Success (Table A2), and we detail causes of—and solutions to—any failures. Alongside these technical outcomes, we were able to successfully record and classify behaviors on the nest during the chick‐rearing period, alongside interspecific interactions, where it was possible to identify other species entering the nest cavity. Details of these behaviors and interspecific interaction observations will be available elsewhere (H. F. R. Hereward, unpublished).

## 3 RESULTS

Across the two breeding seasons, there were 138 camera deployments in 54 different nests, which created a total of 109,183 videos (each 30 s long) (Tables A3, A4 and A3, A4). Of these 138 deployments across both species, 70% of all deployments (*n* = 97) were deemed to be Successful, which equated to 86% of the individual 30‐s videos (94,526, 30 s videos). A further 14% of all deployments (*n* = 20) were deemed to be Partial (usable), which added an additional 13% of usable 30‐s videos (14,595, 30‐s videos) (Tables A3, A4 and A3, A4). Combining both Successful and Partial (usable) deployments and videos together, this equated to a total of 84% usable deployments (*n* = 117) and 99% useable 30‐s videos (109,121, 30‐s videos) (Tables A3, A4 and A3, A4). Partial (usable or nonusable) or total Failures accounted for 30% of deployments and were categorized into troubleshooting and biological issues (Table 1). Solutions to Failures and Partial failures are detailed in Table 2.

**TABLE 1:** Counts of deployment causes of Failure, Partial failure (nonusable), and Partial failure (usable) from all deployments on Praia islet, Graciosa, Azores

	Issues identified	Failure	Partial (nonuseable)	Partial (useable)	Total
Monteiro's storm‐petrel					
Troubleshooting issues	Battery	2	0	2	4
	Humidity, overheating, dislodged connection	4	0	3	7
	Unknown issue causing break in footage	0	0	9	9
	Camera placing	0	0	1	1
Biological issues	Limited movement (adult incubating egg)	0	0	1	1
	Limited movement (egg alone)	0	1	1	2
Madeiran storm‐petrel					
Troubleshooting issues	Humidity, overheating, dislodged connection	9	1	1	11
	Rain or nest empty	0	1	0	1
	PIR sensor connection	0	0	1	1
	Camera placing	0	0	1	1
Biological issues	Nest empty	2	1	0	3
Total		17	4	20	41

Note

Categorized into species (Monteiro's storm‐petrel *Hydrobates monteiroi* and Madeiran storm‐petrel *Hydrobates castro*) and technical troubleshooting and biological issues.

**TABLE 2:** Causes of the Failure and Partial failure deployments during the breeding seasons of both storm‐petrel species, and solutions to address these causes

Broad causes of Failure/Partial failure	Specific causes	Solutions
Trouble‐shooting issues		
Rain, Humidity, overheating, dislodged connection, PIR sensor connection, Break in footage	Isolated islet, where sea spray and rain are frequent throughout the year	Restrict entry/exit holes to camera/powerpack boxes using blue tac/glue at the holes to make it more waterproof Use silica gel sachets in the camera/powerpack boxes to mitigate humidity in the boxes Take the in‐nest camera apart every 6–10 deployments to spend 24−48 hr in a sealed container with silica gel to reduce humidity around the components
	Lifting the camera once deployed sometimes caused connection dislodgement	Avoid moving cameras during deployment Replace cables/kit when worn
Battery	Running out of battery led to no or few recordings	Ensure the battery is fully charged before deployment, if it continues to be a problem consider a larger capacity battery and/or solar panels.
Camera placing	Nest dimensions, including depth, varied	Adjust the camera housing accordingly
Biological issues		
Nest empty	No or limited movement in the nest led to no or few recordings	Absence of recordings indicate that the box is not (yet) being used

Note

The causes are separated into technical trouble‐shooting issues and biological issues.

## 4 DISCUSSION

Here, we have described and demonstrated the successful building and deployment of a bespoke camera that is small, portable, weatherproof, battery‐run, and with PIR motion‐trigger capabilities. This bespoke camera, based on a Raspberry Pi microcomputer, is cheaper or similarly priced to other bespoke cameras of similar build (Prinz et al., 2016; Zárybnická et al., 2016). The poweradd Pilot X7 20,000 mAh powerpack proved to have enough capacity for a 24‐ to 48‐hr deployment if needed (Youngblood, 2020). This deployment duration could be further improved to last for longer per deployment, or to allow for more deployments, for example, by employing the use of camera‐specific solar panels to extend battery life (McBride & Courter, 2019; Nazir, Newey, et al., 2017; Prinz et al., 2016).

In comparison with previous nest box/cavity system designs (e.g., Kalhor et al., 2019; Kallmyer et al., 2019; Prinz et al., 2016; Zárybnická et al., 2016), our camera housing was independent of the nest box design and so completely portable, allowing easy transfer between nests throughout the breeding season, thus allowing us to gain insight into a wider number of individual nesting behaviors as well as avoiding missing out on recordings because individuals did not use an initially targeted nest box (as has occurred previously, e.g., Prinz et al., 2016; Zárybnická et al., 2016).

Despite the increased portability and easy access to download the data, the need to frequently open up the camera housing increased the system's vulnerability to salt spray and humidity, and left parts vulnerable to dislodgement and degradation due to these environmental conditions. Nevertheless, the weatherproofing of the camera housing was generally successful or partially successful (combining “Successful” and “Partial Failure [usable]” footage; 84% usable deployments and 99% of videos usable), which is similar to some previous studies (e.g., 96% of photos usable, McBride & Courter, 2019) and substantially more successful than others (e.g., in Camacho et al., 2017, after 1 month of deployments, 80% of the cameras had ceased to function due to humidity and vandalism; and Kalhor et al., 2019, recorded a 100% deployment success rate, but only 32% of videos were considered of high enough quality to be retained for future analysis). In the present study, ~14% of deployments had troubleshooting issues specifically due to the weather/humidity, particularly in the winter (Madeiran storm‐petrel) breeding season, which was typically cooler and wetter than the summer (Monteiro's storm‐petrel) breeding season (Monteiro & Furness, 1998). This is despite mitigation efforts already employed from previously published papers, including housing as much of the equipment as possible within waterproof casings (McBride & Courter, 2019; Prinz et al., 2016; Youngblood, 2020) and including silica gel packets to reduce humidity within the equipment casing during deployments (Youngblood, 2020). Consequently, some additional waterproofing is suggested alongside the further housing adjustments summarized in Table 2, to aid in reducing these specific failures in future. These mitigations include placing the camera in a box of silica gel between deployments, to reduce the humidity around the components, prior to re‐deployment. The calculated percentage success rates based on the Successful, Partial (usable), Partial (nonusable), and Failure definitions could be used by researchers to estimate how many total successful deployments will be needed to achieve a target sample size.

The present study provides a template for building and programming a bespoke, portable camera paired with a PIR sensor, particularly suitable for use in remote study locations with burrow‐ or cavity‐breeding species, where camera size needs to be minimized and limited power is a constraining factor. Due to its portability and mitigation against salt spray and humidity, this template could be applied to a wide range of different species that utilize cavities, burrows, and artificial nests, or potentially adapted for other wildlife surveillance situations, to monitor behaviors and interspecific interactions, as demonstrated in this study. To further extend the data‐gathering capabilities of these cameras, future additions to this template design could include a microphone to record vocalizations, and temperature and humidity modules to record changes in nest‐specific environmental conditions, for example, to monitor daily, seasonal, and between‐year variations in these variables, or as a comparison between natural and artificial cavities.

## CONFLICT OF INTEREST

We declare we have no conflict of interests.

## AUTHOR CONTRIBUTIONS

**Hannah F. R. Hereward:** Conceptualization (lead); Data curation (lead); Formal analysis (lead); Funding acquisition (equal); Investigation (lead); Methodology (lead); Project administration (equal); Resources (equal); Software (equal); Validation (equal); Visualization (equal); Writing‐original draft (lead); Writing‐review & editing (lead). **Richard J. Facey:** Conceptualization (equal); Methodology (equal); Software (equal); Supervision (equal); Writing‐original draft (equal); Writing‐review & editing (equal). **Alyssa J. Sargent:** Data curation (equal); Methodology (equal); Writing‐review & editing (equal). **Sara Roda:** Data curation (equal); Writing‐review & editing (equal). **Matthew L. Couldwell:** Data curation (equal); Writing‐review & editing (equal). **Emma L. Renshaw:** Data curation (equal); Writing‐review & editing (equal). **Katie H. Shaw:** Data curation (equal); Writing‐review & editing (equal). **Jack J. Devlin:** Data curation (equal); Writing‐review & editing (equal). **Sarah E. Long:** Data curation (equal); Writing‐review & editing (equal). **Ben J. Porter:** Data curation (equal); Writing‐review & editing (equal). **Jodie M. Henderson:** Data curation (equal); Formal analysis (equal); Writing‐review & editing (equal). **Christa L. Emmett:** Formal analysis (equal); Methodology (equal); Writing‐review & editing (equal). **Laura Astbury:** Formal analysis (equal); Methodology (equal); Writing‐review & editing (equal). **Luke Maggs:** Methodology (equal); Resources (equal); Software (equal); Writing‐review & editing (equal). **Sean A. Rands:** Funding acquisition (equal); Project administration (supporting); Supervision (equal); Validation (equal); Visualization (equal); Writing‐review & editing (equal). **Robert J. Thomas:** Conceptualization (equal); Funding acquisition (equal); Methodology (equal); Project administration (supporting); Supervision (lead); Validation (equal); Visualization (equal); Writing‐original draft (equal); Writing‐review & editing (equal).

### OPEN RESEARCH BADGES

This article has been awarded Open Data and Open Materials Badges. All materials and data are publicly accessible via the Open Science Framework at https://doi.org/10.5061/dryad.9w0vt4bfb.

## Data Availability

Deployment data and python scripts archived with Dryad: Hereward, Hannah et al. (2022), Raspberry Pi nest cameras ‐ an affordable tool for remote behavioural and conservation monitoring of bird nests, Dryad, Dataset, https://doi.org/10.5061/dryad.9w0vt4bfb.

## APPENDIX A Detailed part list, program scripts, and extended build instructions

### PART LIST

#### Single‐buy kit for setup

Setting up the camera requires the following single‐purchase kit: a Pimoroni Three Port USB Hub with Ethernet—microB connector, a USB keyboard, a USB mouse, a computer screen (for fieldwork deployments, we recommend a small bespoke screen; the Elecrow 5 Inch Touch Screen HDMI Monitor Small HD 800×480 TFT LCD Display for Raspberry Pi), an HDMI cable with mini‐HDMI adapter, either two micro‐USB cable chargers (for mains power), or two micro‐USB cables with USB‐compatible rechargeable powerpacks (if mains power is not available). Where possible, we recommend setting up cameras using mains power prior to deployment in a remote location, to minimize battery usage in the field. This equipment costs ~£100 GBP (~$133 USD) to allow the construction of multiple cameras prior to deployment.

#### List of parts required to build each camera

The following parts are required to build each camera. Each camera cost a total of ~£86 GBP (~$115 USD) to build. Each numbered part is referred to within the build instructions below:

- *Part 1: Dual USB flash drive, Mini USB to USB‐3.0*. We used a SANDISK Ultra (64 GB), which has a mini‐USB connector on one end of the USB and a USB‐3.0 connector on the other end. This is where the recorded video files were stored. In this study, we used a USB to store video files and found this to be highly successful and aided in smooth transfers of files from cameras after each deployment. This reduced the SD space limitations mentioned in previous papers (McBride & Courter, 2019; Mouy et al., 2020; Prinz et al., 2016; Youngblood, 2020) and, for our deployments, allowed storage of the 3–5 GB of video files created per 24‐ to 48‐hr deployment. However, for those setting up cameras in extreme conditions, the preliminary trials from Mouy et al. (2020) using a USB are important to take into account, as they found the USB‐USB port connection to be fragile and consequently disrupted by vibrations during transport by boat prior to deployment. We suggest labeling each USB flash drive to ensure that each USB drive stays with the same Pi Zero board and to avoid confusion if multiple cameras are being set up and deployed.
- *Part 2: Micro‐SD card*. This SD card acts as the Raspberry Pi computer's hard drive and holds a copy of the Python command scripts. A 16 GB micro‐SD card provided sufficient storage space (we specifically used a NOOBS 16 GB microSD card (version 2.8) as the Raspberry Pi Operating System is pre‐installed). For future users working in remote locations, we would recommend setting up the cameras and cloning the SD cards before the start of fieldwork. This consequently reduces setup time in the field and is especially useful when there is limited or unreliable access to electrical power.
- *Part 3: Raspberry Pi Zero WH (presoldered) board*. The small size of this circuit board allows the camera to be as compact as possible to fit within the nest box. This circuit board does not require soldering (a version without the GPIO header pins attached is cheaper, but requires soldering). In the Python script, the GPIO pins were set to “GPIO layout” (see “nestcam.py”).
- *Part 4: Fisheye camera + infrared LED attachments*. In order to capture the widest possible field of view, and to allow for day and night footage of nests to be recorded, we used a night vision camera module for Raspberry Pi, incorporating a 160° fisheye lens with a standard focus distance of ~15 cm, combined with infrared LED attachments (shop.pimoroni.com).
- *Part 5: Real Time Clock*. To provide an accurate date and time stamp on video recordings, we used a DS1307 RTC Real Time Clock Module Board with additional GPIO pins, powered separately with a LiCB CR1220 3V Lithium Battery Button Cell Battery. Occasionally, we found video files returned with the wrong date and/or time. This was likely due to an insecure connection to the Real Time Clock, or because the separate button battery was running low, causing the Real Time Clock date and time to reset itself. This is easily fixed between deployments, by replacing the button battery (or the whole component), and then repeating the Real Time Clock setup as described below. For buffering against such technical failures, we recommend taking several sets of spare components into the field to allow for smooth and quick fixes if changes are needed.
- *Part 6: Passive Infrared (PIR) sensor + 3 female–female cables*. To enable the camera to record after detection of motion, a pre‐assembled PIR sensor was used (Figure 2a–d). This method assumed that a change in infrared detection would indicate that motion of an animal had occurred within the field of view.
- *Part 7: Off clicker + 2 female–female cables*. To allow for correct shutting down of the Pi Zero board in the field, a “shutdown” script was written (see “shutdown.py”). On the switch end of the female–female cables, the black covers were removed for easier attachment of the switch (Figure 1e–g).
- *Part 8: Heat sink*. One heat sink was added to each Raspberry Pi Zero board, to reduce the risk of overheating.
- *Part 9: Mini‐USB‐3.0 USB connector cable + powerpack*. To power the Pi Zero board, a 1‐m‐long mini‐USB‐3.0 connector cable was connected to a Poweradd Pilot X7 20,000 mAh portable powerpack (these powerpacks typically powered the camera setup for 24–48 hr).

#### Equipment housing

For our study system, the design of the equipment housing was an important consideration, given the high relative humidity and salt spray, and the pre‐existing artificial nest box dimensions. As a result, we used two sizes of plastic container to house the equipment: a smaller one for the camera (Lock & Lock HPL805 Stackable Airtight Container Rectangular 180 ml, Plastic, Clear, 11 × 8.9 × 4.9 cm) and a larger one for the powerpack (Lock & Lock 800 ml Food Container Rectangular Lunch Box HPL816, 13.7 × 5.3 × 20.8 cm). Holes to accommodate the camera wiring were drilled into appropriate places. Blue tac (Bostik) and glue (such as PVC pipe adhesive) were used to seal the gaps in the drilled holes where necessary, reducing the likelihood of water entry, and at least one 1‐g silica gel sachet was also placed inside each of the sealed boxes to help reduce humidity around the equipment.

#### Camera mounts

To mount the cameras on top of the nest rim, but underneath the lid, we cut a hole from the center of a round cork board (25 cm diameter, 1 cm thick) into which the waterproof camera box base slotted. We then attached (using thin garden wire) an additional thinner cork board square (cut from a 22‐cm‐diameter, 0.6‐cm‐thick cork board) with camera, IR LED and PIR sensor holes to support these (Figure 2a–d). The equipment housing and camera mounts cost an additional ~£23 GBP (~$31 USD) per camera.

#### Program scripts and associated files

Five python files can be found in the archived data repository (Hereward et al., 2022). Two are python scripts that were used to run the camera and shutdown option (“nestcam.py” and “shutdown.py”). The other three files are details of the command lines to be used in the Raspberry Pi “terminal,” which assist in the camera setup described below (“script for RaspPi terminal_RTC.py,”“script for RaspPi terminal_runonboot.py,” “script for RaspPi terminal_usb.py”).

#### Step‐by‐step instructions for building the camera

Using the parts described above, and each of the scripts provided, we suggest setting up the camera in this order:

1. Before beginning to build the camera, plug the uniquely labeled USB (Part 1) into a computer and copy the five python files (see archived data repository) onto the USB. Note that in any future connections of the USB to the computer it will suggest “fixing a bug problem”—do not select this option as it will reformat the USB.
2. Install onto the microSD card (Part 2) the Raspberry Pi Operating System, which is downloadable (with installation instructions) from: www.raspberrypi.org/downloads/raspbian/ (if you have bought a NOOBS microSD then the system is pre‐installed so you can skip this step).
3. Insert the microSD card (Part 2) into the Pi Zero board (Part 3) and connect the Pimoroni Three Port USB Hub to the Pi Zero “USB” port. Connected to this three‐port hub should be the USB (Part 1), keyboard and mouse. Then connect the screen using the HDMI cable with mini‐HDMI adapter.
4. Once the above items have been connected, only then connect the power source for the screen and Pi Zero board, using USB cables to mains power or USB cables to powerpacks.
5. Configure the SD card by following these dropdown menus: pi → Preferences → Rasp.pi configuration – interfaces – enable…. Enable: “camera,” “SSH,” and “I2C.”
6. In the folder window, find the Pi folder (home → Pi) and create two folders, “scripts” and “usb” (this creates folders on the microSD card—note the use of all lowercase letters in folder names).
7. From the USB, copy over the scripts into the new “scripts” folder.
8. At this point, the USB is recognized in the folder: home → media → USB, but the next step is to “mount” the USB so that the USB is always given the same name/location and so that the data can be written to this location (each USB has a unique code hence the importance of labeling each USB so it remains with the same Pi Zero board after “mounting” it). Here, we set the new USB folder in: home → Pi → usb ‐ this is the folder where the USB will then always open. To do this, follow the step‐by‐step guide in the "script for RaspPi terminal_usb.py."
9. Once these steps are completed, the rest of the camera can be built up on the Raspberry Pi board following these steps:
  - Turn off the board (Pi → shutdown).
  - Add the camera (Part 4) to the Camera Serial Interface port on the Raspberry Pi board.
  - The Real Time Clock (RTC; Part 5) is then placed on the GPIO pins 1–10 (GPIO pins are numbered starting from pin 1 at the SD card end).
  - The pre‐assembled PIR sensor (Part 6) is then added to the additional pins on the Real Time Clock using three female‐female cables. The PIR sensor “VCC” pin is connected to the Real Time Clock “5V” pin and then the two “GND” pins are connected together. Finally, “OUT” on the PIR sensor is connected to GPIO17 (pin 11) on the Pi Zero board (Figure 1a–d). The sensor settings (time and sensitivity) are then altered using the settings; Time = min, sensitivity = 90° to min (Figure 1a).
  - The “Off” switch (Part 7) is added to pins 39 (GND) & 40 (GPIO 21) (i.e., the end GPIO pins) using two female–female cables (Figure 1e–g).
  - Add the Heat sink (Part 8); remove the peel‐off‐sticker and place onto the chip on the Raspberry Pi board (Part 3).
  - Then reconnect the power via the USB connector cable.
10. Next, configure the Real Time Clock to run on the correct time and date. To do this, follow the step‐by‐step guide in the “script for RaspPi terminal_RTC.py.”
11. Before configuring the terminal so that the scripts run on boot (i.e., run automatically when power is connected), it is useful to check that the camera script is working. Open Pi → Programming → Python 3 (IDLE) → file → open → scripts → “nestcam.py” and press F5 to run the script. Pressing “shift and F6” stops the script running. You will notice that the “nestcam.py” is scripted to print the word “idle” when the camera is off and “recording” when the camera is recording. This is displayed on the python shell output screen, and aids in testing the camera before deployment.
12. Once you have checked that the camera is working correctly, add the “nestcam.py” and “shutdown.py” scripts to the “bootup” so that they will run when it is connected to power; see the step‐by‐step guide in the “script for RaspPi terminal_runonboot.py.”
13. When all of the components are assembled (Figure 1h) and configured, the camera is ready to deploy in the field. Disconnect the HDMI cable + mini‐HDMI adapter, Pimoroni Three Port USB Hub (with keyboard and mouse) and connect the USB (Part 1) directly to the “USB” port on the Raspberry Pi board (Part 3).
14. Prior to deployment the camera needs to be fitted into the weatherproof housing as described above.
15. Finally, when ready to turn the camera on, connect a Mini USB 3.0 USB connector cable (Part 9) to the “power in” port on the Raspberry Pi board (Part 3) and the powerpack (Part 9).

**TABLE A1:** Cost breakdown by component, for the single‐purchase kit requirements, camera, and housing used in this study (costing as of July 2020)

Equipment	Quantity	Single‐purchase kit	Per camera	Per housing
Elecrow 5 Inch Touch Screen for Raspberry Pi	1	£32.99		
HDMI cord	1	£3.50		
Mini HDMI converter	1	£2.96		
Micro‐USB cable + charger (e.g., Raspberry Pi 3 Power adapter UK/EU 5V 2.5A OR using micro‐USB ‐ USB cable + powerpack already acquired to run the boards)	2	£16.99		
Wired USB keyboard + USB mouse	1	£28.98		
Pimoroni Three Port USB Hub with Ethernet ‐ microB connector	1	£9.90		
Spare Lock & Lock 800ml Food Container Rectangle Container Lunch Box HPL816	1	£4.00		
Silica gel (20g packet)	5	£10.00		
Total for single kit requirements		£109.32		
Raspberry Pi Zero WH (presoldered) board	1		£13.02	
Real Time Clock (RTC)	1		£9.99	
PIR sensor (connected using Female‐Female jump leads)	1		£3.50	
160° fish eye lens with infrared attachments (+zero lead)	1		£20.00	
NOOBS SD card (with micro SD card)	1		£9.00	
Mini USB to USB 3.0 USB	1		£11.99	
Off switch/clicker	1		£0.03	
Poweradd Pilot X7 20,000 mAh portable powerpack	1		£15.99	
Mini USB 3.0 USB connector cable	1		£2.30	
Heat sink	1		£0.50	
Female‐female jump leads × 3 (for motion sensor), ×2 (for switch) (0.075p per lead)	5		£0.38	
Total for camera			£86.70	
Lock & Lock HPL805 Stackable Airtight Container Rectangular 180 ml	1			£3.50
Lock & Lock 800 ml Food Container Rectangle Container Lunch Box HPL816	1			£4.00
Cork board ‐ 25 cm diameter round, 1 cm thick	1			£7.00
Cork board ‐ 22 cm diameter round, 0.6 cm thick	1			£2.00
Garden wire diameter ~1.2 mm (small amount needed from large reel)	1			£6.00
Silica gel (1 g packet)	1			£0.08
Total for housing				£22.58
Total for the camera + housing	£109.27			
Overall total	£218.59			

Note

Prices given in GBP £.

**TABLE A2:** Description of the Success, Partial failure, or Failure of each in‐nest camera deployment

Type of deployment success/failure	Description
Failure	Where only 0–2 videos recorded
Partial failure (nonusable)	When more than two videos were recorded but in total less than 1 hr was recorded
Partial failure (usable)	When there was an unexpected interruption in the footage but there was more than 1 hr of footage recorded (e.g., caused by loss of battery power, technical faults, or a break in footage despite movement still occurring in the nest due to an adult or chick being present)
Success	Continuous footage with no known interruptions (allowing for anticipated breaks between footage when no movement was detected)

**TABLE A3:** Number of deployments, number of 30‐s videos created per deployment, sum, maximum, mean, and standard error of the hours of footage recorded

	Deployment outcome	No. of deployments	No. of videos	Hours of footage recorded			
				Sum	Max	Mean	SE
Monteiro's storm‐petrel	Failure	6	3	0.0	0.0	0.0	0.0
	Partial (nonuseable)	1	6	0.1	0.1	0.1	0.0
	Partial (useable)	17	14,086	117.4	22.0	6.9	1.4
	Success	56	52,118	434.3	14.9	7.8	0.5
Subtotal		80	66,213	551.8	37.0	14.7	
Madeiran storm‐petrel	Failure	11	10	0.1	0.0	0.0	0.0
	Partial (nonuseable)	3	43	0.4	0.2	0.1	0.0
	Partial (useable)	3	509	10.7	6.5	3.6	1.5
	Success	41	42,408	353.4	17.5	8.6	0.5
Subtotal		58	42,970	364.5	24.2	12.3	
Total		138	109,183	916.3	22.0	6.6	0.4

Note

Categorized into if the deployment was a Failure, Partial failure (nonusable), Partial failure (usable), or Success both species: Monteiro's storm‐petrel *Hydrobates monteiroi* and Madeiran storm‐petrel *Hydrobates castro*, from their respective breeding seasons (summer 2019 and winter 2019–2020, respectively) on Praia islet, Graciosa, Azores.

**TABLE A4:** Number of Successful and Partial failures (usable) deployments categorized by the duration of the footage obtained based on start and end times of the footage (1–12 hr, 12–24 hr, 24+ hr) for both species: Monteiro's storm‐petrel *Hydrobates monteiroi* and Madeiran storm‐petrel *Hydrobates castro* from their respective breeding seasons (summer 2019 and winter 2019–2020, respectively) on Praia islet, Graciosa, Azores

Deployment outcome	Storm‐petrel species	Duration of footage			
		1–12 hr	12–24 hr	24+ hr	Total
Successful	Monteiro's	7	30	19	56
	Madeiran	0	37	4	41
	Subtotal	7	67	23	97
Partial (useable)	Monteiro's	7	2	8	17
	Madeiran	1	0	2	3
	Subtotal	8	2	10	20
	Total	15	69	33	117
